# Trajectories and single-particle tracking data of intracellular vesicles loaded with either SNAP-Crb3A or SNAP-Crb3B

**DOI:** 10.1016/j.dib.2016.04.058

**Published:** 2016-04-29

**Authors:** Jan Peter Siebrasse, Ivona Djuric, Ulf Schulze, Marc A. Schlüter, Hermann Pavenstädt, Thomas Weide, Ulrich Kubitscheck

**Affiliations:** a Institute of Physical and Theoretical Chemistry, Rheinische Friedrich-Wilhelms University Bonn, Wegelerstraße 12, 53115 Bonn, Germany; b Internal Medicine D, Molecular Nephrology, University Hospital of Muenster, Germany

## Abstract

Using a combined approach of pulse chase labeling and single-particle tracking of Crb3A or 3B loaded vesicles we collected trajectories of different vesicle population in living podocyte cells and evaluated statistically their different mobility patterns. Differences in their intracellular mobility and in their directed transport correspond well to the role of Crb3A and 3B in renal plasma membrane sorting (Djuric et al., 2016) [1].

**Specifications Table**

**Table:** 

Subject area	*Biophysics, Cell Biology*
More specific subject area	*Single*-*particle-tracking, vesicle trafficking*
Type of data	*Graph, figure*
How data was acquired	*Single Molecule Microscopy, Single*-*particle-tracking*
Data format	*Analyzed*
Experimental factors	*SNAP*-*tag based fluorescence labelling in live cells*
Experimental features	*Human podocytes (AB8) stably expressing either Crb3A or Crb3b fused with the SNAP tag were fluorescence labelled using a respective SNAP tag dye*
Data source location	*University of Bonn, Germany*
Data accessibility	*Data is within this article*

**Value of the data**

• Renal plasma membrane proteins Crb3A and Crb3B can be assigned to different vesicle subpopulations.
• The different membrane proteins can be used as distinct vesicle marker to identify and track different vesicle populations.
• Differences in mobility pattern and trafficking dynamics might be related to intracellular redistribution of Crb3A and Crb3B and their respective plasma membrane portion.

## 1 Data

**Fig. 1:**
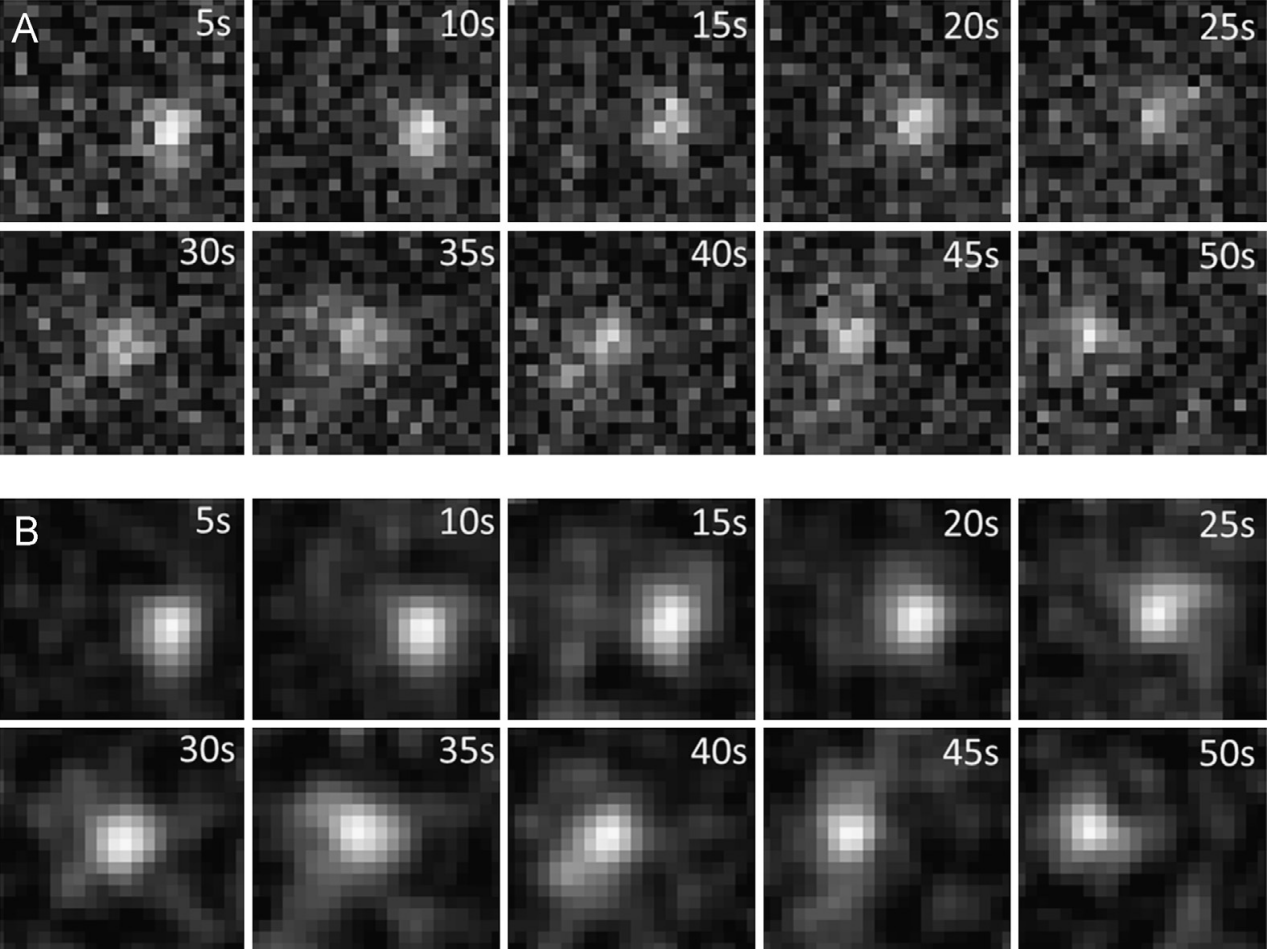
(A) Image raw data. Pixel size is 95.2 nm, movies of 128×128 pixels were acquired at a frame rate of 10 Hz. Shown are sub frames of 22×20 px (2.0×1.9 µm²) with a single Crb3A-positive vesicle (the corresponding trajectory is depicted in Fig. 2A. (B) Filtered image sequence after background subtraction (Rolling ball 50 px), contrast enhancement (0.1% saturated pixel) and smoothing (Gauss filter with Kernel size=1 pixel) using ImageJ [4].

**Fig. 2:**
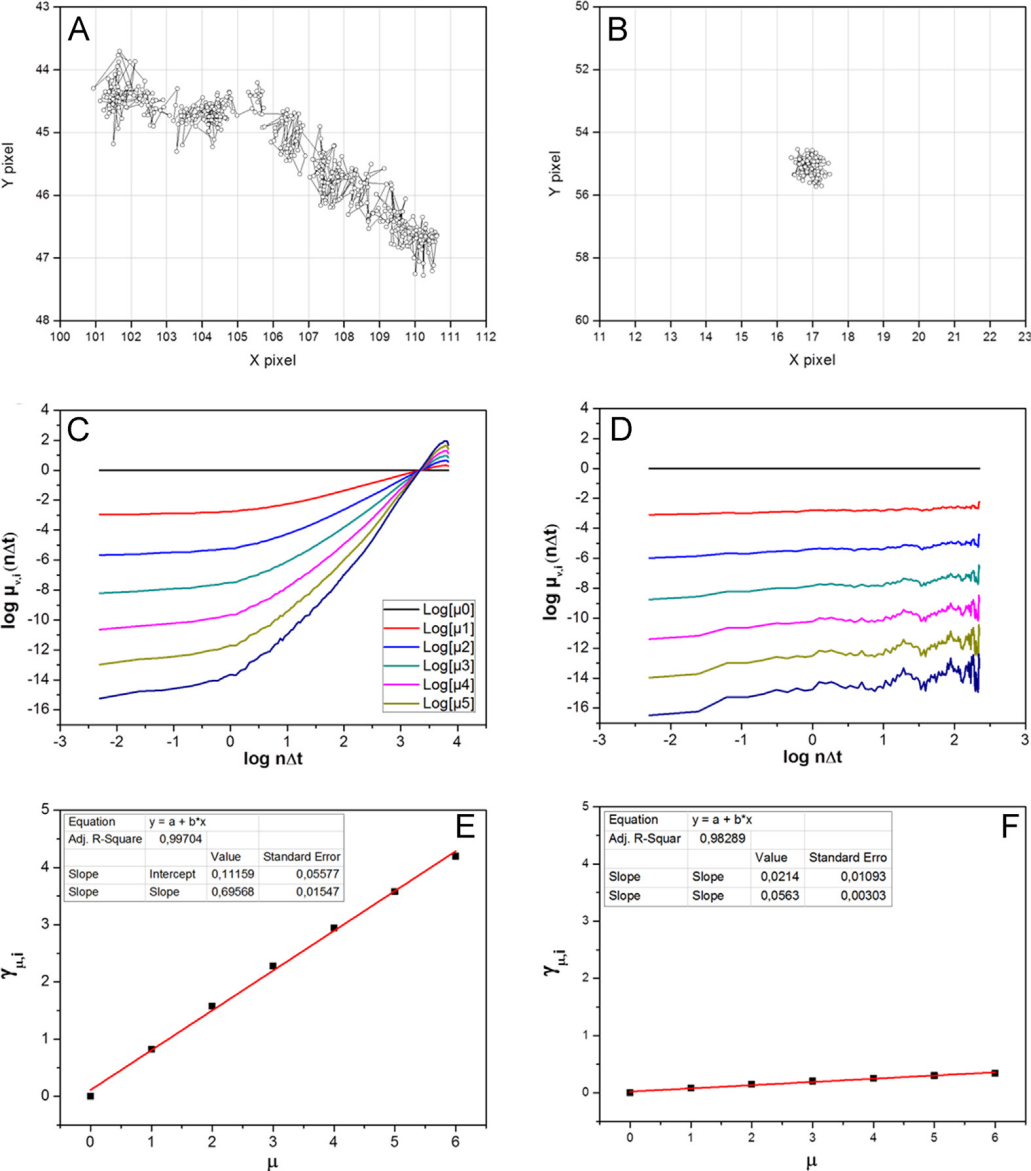
Motion analysis based on trajectories derived from Crb3A movies (A, B), respective plots of (log *µ*_n_(*n*Δ*t*) vs. log *n*Δ*t*) (C, D) and their moment scaling spectrum (MSS; E, F) according to [2], [3].

**Fig. 3:**
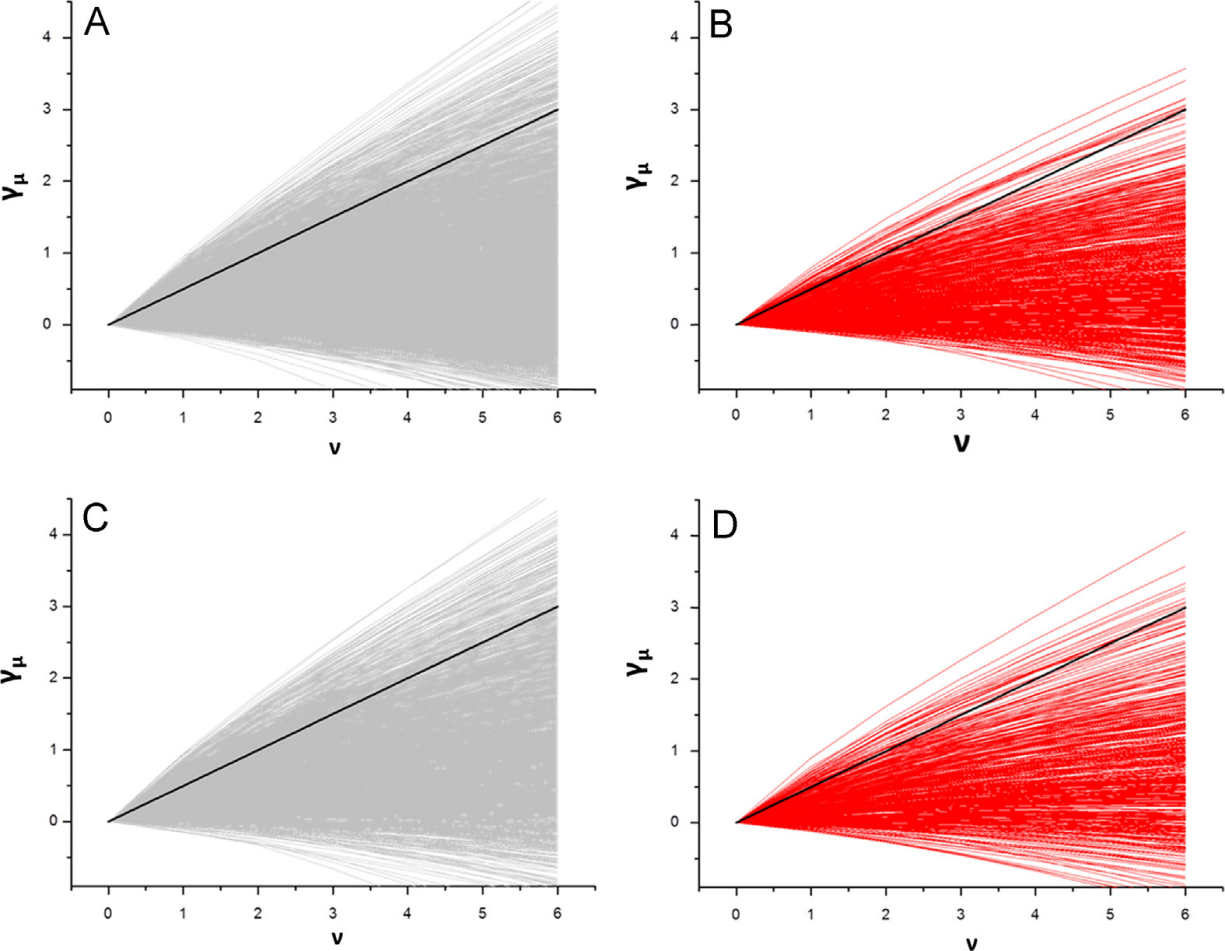
Moment scaling spectrum (MSS) from many Crb3A (A) and Crb3B (C) trajectories (solid black lines included to indicate pure diffusion). Respective MSS plots after Nocodazole treatment for Crb3A (B) and Crb3B (D).

Single vesicles loaded with different membrane proteins fused to a SNAP-tag were tracked in living cells after fluorescence labeling (Fig. 1). From single trajectories the *momentum scaling spectrum* was computed and its slope calculated [2], [3] (Fig. 2). The slopes from many different trajectories were collected (Fig. 3A and C) and compared to the slope of trajectories after respective drug treatment (Fig. 3B and D). The distinct mobility patterns of Crb3A versus Crb3B vesicles can be best visualized by a histogram of all *S*_MSS_ (slope of the momentum scaling spectrum) as it is shown in Djuric et al. [1].

## 2 Experimental design, materials and methods

### 2.1 Cell culture and SNAP-tag labeling

The SNAP-tag fusion protein expressing AB8 cells were seeded on cover slides and incubated in standard RPMI 1640 medium containing 10% FCS and supplements and 1% antibiotics (Pen/Strep). Directly before the measurements the cells were incubated with the fluorescent SNAP-tag substrate (BG594) according to the manufacturer׳s protocol. After incubation free dye was removed by washing several times.

### 2.2 Live cell Imaging and vesicle tracking

Cells were imaged using an inverted custom-built single-molecule microscope based on an Axiovert 200TV (Zeiss, Jena, Germany) equipped with a 63×NA 1.2 water immersion objective lens [5]. For excitation the 532 nm laser line of a Cobolt Laser (Cobolt Dual Calypso, 100 mW) was used and movies were recorded with an electron multiplying CCD camera (iXon BI DV-860, Andor Technologies, Belfast, Ireland) at 10 Hz frame rate. Tracking of single vesicles was done with ImageJ and the Particle Tracker 2D/3D plugin [6] using the unfiltered data.

### 2.3 Analysis of vesicle mobility

We followed an approach from Ewers et al. [2] who studied the different mobility patterns of virus like particles during infection. The *x*/*y* coordinates of the trajectories were exported to Origin8 (OriginLab Corp., Northhampton, USA) and further analyzed used a custom-built OriginC script, which allowed automated calculation of the so called momentum scaling spectrum (MSS) and its slope, *S*_MSS_. For each trajectory *i* the moments of displacement (*µ*_ѵ,i_) were calculated for *ѵ*=1, …, 6 as a function of time according to:

$${µ}_{ѵ,i}\left(n∆t\right)=\frac{1}{M_{i}-n}\sum_{m=0}^{M_{i}-n-1}{|x_{i}\left(m+n\right)-x_{i}(m)|}^{ѵ}$$

Here, *x*_i_(*n*) designates the position vector of trajectory *i* at time *n*Δ*t*, where Δ*t* is the inverse frame rate and *n* a specific frame number: *n*=0, 1, …, *M*_i_−1 (*M*_i_ is the trajectory length). For *ѵ*=2 this matches with the “classical” mean square displacement analysis. Using all momenta allowed construction of the MSS for each single trajectory [2], [3]. Therefore *µ*_ѵ,i_(*n*Δ*t*) was plotted versus *n*Δ*t* in a double logarithmic plot (Fig. 1C and D). From these plots the so called *scaling moments γ*_ѵ,i,_ can be derived assuming each moment *µ* depends on the time shift according to *µ*_ѵ_(*n*Δ*t*)~*n*Δ*t*^γѵ^ [2], [3]. Plotting *γ*_ѵ_ versus *ѵ* will finally give the respective MSS (Fig. 2E and F). The MSS will show a straight line through the origin and its slope is an excellent measure for the type of movement. In case of free, unhindered diffusion the slope is 0.5, a slope >0.5 indicates directed motion or active transport, while a slope <0.5 implies retardation or immobilization.
